# Auditory, Cognitive, and Linguistic Factors Predict Speech Recognition in Adverse Listening Conditions for Children With Hearing Loss

**DOI:** 10.3389/fnins.2019.01093

**Published:** 2019-10-15

**Authors:** Ryan W. McCreery, Elizabeth A. Walker, Meredith Spratford, Dawna Lewis, Marc Brennan

**Affiliations:** ^1^The Audibility Perception and Cognition Laboratory, Boys Town National Research Hospital, Omaha, NE, United States; ^2^Pediatric Audiology Laboratory, Department of Communication Sciences and Disorders, University of Iowa, Iowa City, IA, United States; ^3^Amplification and Perception Laboratory, Department of Special Education and Communication Disorders, University of Nebraska, Lincoln, NE, United States

**Keywords:** children, hearing loss, noise, reverberation, speech recognition, hearing aids

## Abstract

**Objectives:** Children with hearing loss listen and learn in environments with noise and reverberation, but perform more poorly in noise and reverberation than children with normal hearing. Even with amplification, individual differences in speech recognition are observed among children with hearing loss. Few studies have examined the factors that support speech understanding in noise and reverberation for this population. This study applied the theoretical framework of the Ease of Language Understanding (ELU) model to examine the influence of auditory, cognitive, and linguistic factors on speech recognition in noise and reverberation for children with hearing loss.

**Design:** Fifty-six children with hearing loss and 50 age-matched children with normal hearing who were 7–10 years-old participated in this study. Aided sentence recognition was measured using an adaptive procedure to determine the signal-to-noise ratio for 50% correct (SNR50) recognition in steady-state speech-shaped noise. SNR50 was also measured with noise plus a simulation of 600 ms reverberation time. Receptive vocabulary, auditory attention, and visuospatial working memory were measured. Aided speech audibility indexed by the Speech Intelligibility Index was measured through the hearing aids of children with hearing loss.

**Results:** Children with hearing loss had poorer aided speech recognition in noise and reverberation than children with typical hearing. Children with higher receptive vocabulary and working memory skills had better speech recognition in noise and noise plus reverberation than peers with poorer skills in these domains. Children with hearing loss with higher aided audibility had better speech recognition in noise and reverberation than peers with poorer audibility. Better audibility was also associated with stronger language skills.

**Conclusions:** Children with hearing loss are at considerable risk for poor speech understanding in noise and in conditions with noise and reverberation. Consistent with the predictions of the ELU model, children with stronger vocabulary and working memory abilities performed better than peers with poorer skills in these domains. Better aided speech audibility was associated with better recognition in noise and noise plus reverberation conditions for children with hearing loss. Speech audibility had direct effects on speech recognition in noise and reverberation and cumulative effects on speech recognition in noise through a positive association with language development over time.

## Introduction

Children spend a considerable amount of time listening in environments with suboptimal acoustics, including high levels of background noise and reverberation (Knecht et al., 2002; Crukley et al., 2011). Because noise and reverberation are ubiquitous, auditory learning, and socialization frequently occur in conditions with an acoustically degraded speech signal. The ability to understand degraded speech is an important developmental skill that does not reach full maturity until adolescence in children with typical hearing (Johnson, 2000; Corbin et al., 2016). The protracted developmental time course for speech recognition in adverse listening conditions in typically developing children has been attributed to the parallel maturation of cognitive and linguistic skills (Sullivan et al., 2015; McCreery et al., 2017; MacCutcheon et al., 2019).

Children with hearing loss face even greater challenges than their peers with typical hearing for understanding speech in adverse acoustic environments. Noise and reverberation frequently co-occur in classrooms and other listening environments experienced by children (Klatte et al., 2010). Whereas, some children with hearing loss who have well-fitted hearing aids or cochlear implants can understand speech in quiet as well as their peers with normal hearing (McCreery et al., 2015), very few children with hearing loss reach comparable levels of performance as their peers with normal hearing in noise (Goldsworthy and Markle, 2019) or reverberation (Neuman et al., 2012). The persistence of speech recognition deficits for children with hearing loss even after access to the signal has been restored with amplification raises questions about the mechanisms that affect the ability to understand degraded speech in everyday listening environments. The main goal of this study was to examine the factors that predicted individual differences in speech recognition in noise and in noise with reverberation by children with hearing loss.

The loss of audibility associated with hearing loss is a primary contributor to difficulties understanding speech in noise or reverberation among children with hearing loss. Blamey et al. (2001) demonstrated that speech recognition for children with hearing loss was strongly related to the child's pure tone average threshold with poorer recognition for children with greater degrees of hearing loss. Children with hearing loss who have better aided detection thresholds for pure tones also had better open-set word recognition (Davidson and Skinner, 2006). However, detection thresholds in quiet may not reflect individual differences in speech recognition in noise, so more recent studies have attempted to use measures of aided speech audibility at conversational levels as a predictor. Speech audibility is often quantified using the Speech Intelligibility Index (SII; ANSI S3.5-1997), which estimates the proportion of the long-term average speech spectrum that is audible. Because the SII directly measures the audibility of speech for an individual, it can be considered a more accurate measure for predicting speech recognition than thresholds from the audiogram. The degree to which hearing loss limits speech audibility has been explored as a predictor of unaided (Scollie, 2008) and aided (Stiles et al., 2012; McCreery et al., 2015, 2017) speech recognition for children. In general, studies have found that children with hearing loss who have greater aided audibility for speech have better aided speech recognition in quiet (McCreery et al., 2015) and in noise (McCreery et al., 2017; Walker et al., 2019).

Mixed findings from the adult literature make predicting the effects of amplification on speech recognition in noise and reverberation for children with hearing loss more challenging. In one study, adults performed better in reverberation with wide-dynamic range compression (WDRC) amplification compared to linear amplification on a sentence recognition task (Shi and Doherty, 2008), suggesting that the increased audibility that occurs with WDRC compared to linear amplification may have enhanced listeners' speech recognition in reverberation. In contrast, Reinhart and Souza (2016) found that recognition for adults with hearing loss in reverberation was best when the release times for the amplitude compression were slower and more linear, as faster compression release times resulted in greater distortion of the temporal envelope of the speech signal. Children's hearing aids are often fitted to optimize speech audibility using WDRC (Scollie et al., 2005), but children are also likely to be more susceptible than adults to distortions of temporal and spectral cues in the speech signal (Hall et al., 2012). Thus, the effects of maximizing audibility with amplification on speech recognition in noise and reverberation for children remain difficult to predict without being directly examined.

Multiple studies have demonstrated that the addition of reverberation negatively affects speech recognition in school-age children with normal hearing (Neuman and Hochberg, 1983; Bradley and Sato, 2008; Wróblewski et al., 2012). In a study of children with normal hearing, Nabelek and Robinson (1982) reported that children required up to 20 dB higher sensation level to reach similar levels of performance as adults when listening in reverberation. A later study by Johnson (2000) found that while the developmental trajectory for speech recognition in noise and reverberation for children with normal hearing often did not reach adult levels of performance until the teenage years, the sensation level did not affect performance across age. Thus, the effects of increasing audibility on speech recognition in noise and reverberation for children have been mixed. However, speech recognition in listening conditions with noise and reverberation for children with hearing loss has not been widely studied. Forty years ago, Finitzo-Hieber and Tillman (1978) conducted what remains one of the only examinations of the effects of noise and reverberation on children with hearing loss using hearing aids. While all children had poorer speech recognition in degraded conditions, the combined effects of noise and reverberation created disproportionate difficulty understanding speech for the children with hearing loss. However, the children with hearing loss in the Finitzo-Hieber and Tillman study used monaural, linear amplification. Also, because the study was conducted prior to the development of hearing aid verification methods, the level of audibility provided by the hearing aids for the children in that study was not specified. The implications of the results for children with hearing loss who use bilateral hearing aids with WDRC are difficult to generalize from this study.

Cognitive and linguistic skills are also likely to support speech recognition in noise and reverberation for children with hearing loss. The Ease of Language Understanding model (ELU; Rönnberg et al., 2013) is a model of language processing that suggests that listeners rely on their knowledge of language and cognitive skills like working memory and attention to understand speech in degraded conditions. The predictions of the ELU model that children with greater working memory capacities have better speech in noise than peers with reduced working memory capacities have been confirmed by some previous studies of speech recognition in noise for children with normal hearing (Stiles et al., 2012; Sullivan et al., 2015; McCreery et al., 2017; MacCutcheon et al., 2019). However, work by Magimairaj et al. (2018) did not find an association between language or working memory and sentence recognition in babble noise for children with normal hearing. The ELU model has also been extended to predict speech recognition in children with hearing loss (McCreery et al., 2015). Children with better working memory and language skills often have better speech understanding in noise than children with poorer working memory and language. Based on the predictions of the ELU model, it is reasonable to predict that cognitive and linguistic skills would be helpful for listening to speech degraded by noise and reverberation.

One potential mechanism that explains the link between cognitive and linguistic skills and listening in noise and reverberation is related to children's increased susceptibility to informational masking (Brungart et al., 2001; see Leibold and Buss, 2019 for a review). Reverberation is a particularly challenging masking signal because the reverberant signal can cause energetic masking, where the energy of the reverberant signal overlaps with the target signal in the auditory system. In addition, reverberation contains speech-like spectral and temporal cues that are similar to speech and create uncertainty about the stimuli, which are both characteristics of informational masking (Durlach et al., 2003). Children are also less likely to benefit from temporal fluctuations in a masker than are adults (Hall et al., 2012), which has been attributed to the development the development of temporal processing. To date, few studies have examined the cognitive and linguistic contributions to the development of informational masking. However, some evidence from adults suggests that listeners with better working memory skills may be less susceptible to distortions of the auditory signal from amplification or hearing loss (see Akeroyd, 2008, for a review). Other researchers have suggested that increased susceptibility to informational masking among children may be related to difficulty segregating the target signal from the masking signal (Leibold et al., 2016). Greater susceptibility to informational masking in children has been attributed to deficits in auditory attention (Allen and Wightman, 1995; Corbin et al., 2016), but the effects of individual attention skills on conditions that produce informational masking have not been directly studied in children to our knowledge.

Potential interactions may exist between amplification and linguistic and cognitive skills that could influence the relationship of these factors with speech recognition in noise and reverberation. Recent evidence suggests that children who have better audibility through their hearing aids not only have better speech recognition (McCreery et al., 2015), but also have stronger language skills (Tomblin et al., 2014, 2015). This relationship between audibility and language development suggests that audibility has immediate effects related to a listener's access to the acoustic signal and cumulative effects related to its long-term influence on language. These relationships could make it difficult to disambiguate the effects of audibility related to access to the speech signal from the cumulative effects of audibility on language skills that are likely to support listening in noise and reverberation. Mediation models (Baron and Kenny, 1986) have been used to examine the pattern of associations between outcomes and predictors that are inter-related. A recent study by Walker et al. (2019) used a mediation analysis to determine if the effects of audibility on speech recognition for a gated word recognition task were direct or mediated by the relationship between audibility and language skills. The results suggested that audibility was related to both language and gated speech recognition, supporting both immediate, and cumulative influences of audibility. A similar approach was used to attempt to disambiguate that complex relationship in the current study.

The overall goal of this study was to examine factors that predicted individual differences in aided speech recognition for children with hearing loss and a group of children with typical hearing matched for age and socioeconomic status (indexed by maternal education level). Three research questions were examined:

- Does listening in noise and reverberation present additional challenges to children with hearing loss compared to peers with normal hearing? Based on previous research, we predicted greater difficulty listening in noise and reverberation for children with hearing loss than peers with normal hearing.
- Consistent with the ELU model, do linguistic and cognitive abilities predict individual differences in speech recognition in noise and reverberation for children with normal hearing and children with hearing loss? We predicted that our results would be consistent with the ELU model in that children with stronger working memory, language, and auditory attention skills would have better speech recognition in noise and reverberation than children with poorer skills in these domains.
- Does aided speech audibility have a direct relationship with speech recognition in noise and reverberation or is the relationship mediated by the child's language skills? Based on previous research, we anticipated that the relationship between audibility and aided speech recognition in noise and reverberation would include direct and mediated paths.

## Method

### Participants

Children with normal hearing (*n* = 50) and children with mild-to-severe hearing loss (*n* = 56) participated in the experiment. Children were recruited from research centers at Boys Town National Research Hospital (Omaha, Nebraska) and the University of Iowa (Iowa City, Iowa) and the surrounding areas as part of a longitudinal study of developmental outcomes for children with bilateral mild-to-severe hearing loss. Table 1 shows the demographic characteristics of the children in the sample. Data collection occurred during the summer following either 1st or 3rd grade when children were 7–9 years-old. All children were from homes where spoken English was the primary language and did not have other diagnosed developmental conditions at the time of enrollment in the study. All 56 of children with hearing loss wore bilateral hearing aids. The study was approved by the Institutional Review Board of Boys Town National Research Hospital.

**Table 1:** Participant characteristics.

**Group**	**Children with normal hearing**	**Children with hearing loss**	**Statistical tests**
Number	50	56	
Sex	Female = 23, Male = 27	Female = 23, Male = 33	*X*^2^ = 1.33, *p* = 0.62
Maternal education level	15.5 years of education	15.3 years of education	*t* = 1.12, *p* = 0.75
Grade	1st grade = 32; 3rd grade = 18	1st grade = 28; 3rd grade = 28	
Age (Years)	1st grade—M = 7.5 (*SD* = 0.4); 3rd grade–M = 9.0 (*SD* = 0.35)	1st grade—M = 7.5 (*SD* = 0.6); 3rd grade M = 9.0 (*SD* = 0.4)	*t* = 0.07, *p* = 0.96 *t* = 0.01, *p* = 0.98
PPVT standard score	Mean = 112.7	Mean = 111.6	*t* = 1.08, *p* = 0.35
NEPSY attention scaled score	Mean = 9.1	Mean = 9.0	*t* = 0.06, *p* = 0.93
AWMA OOO standard score	Mean = 110.9	Mean = 111.7	*t* = 1.24, *p* = 0.18
Age of confirmation of hearing loss	N/A	Mean = 12.7 months, Median = 3 months	
Age of HA fitting	N/A	Mean = 16.4 months Median = 6 months	
Better-ear PTA	N/A	Mean = 45.6 dB HL Median = 44 dB HL	
Better-ear aided SII	N/A	Mean = 0.77 Median = 0.83	
Average hours of HA use per day	N/A	Mean = 9.88 Median = 10.5	
RMS error		Mean left ear = 5.09; Mean right ear = 5.27	

*PPVT, Peabody Picture Vocabulary Test; NEPSY Attention, NEPSY Auditory Attention subtest; AWMA OOO, Automated Working Memory Assessment Odd-One-Out subtest; HL, Hearing Level; PTA, Pure-tone average hearing thresholds at 500, 1,000, 2,000, and 4,000 Hz; SII, Speech-intelligibility index for average speech (60 or 65 dB SPL) at one meter with hearing aids; Hearing aid use based on hearing aid data logging or parent report. RMS error is the geometric mean of the deviations of the hearing aid output from prescriptive target at 500, 1,000, 2,000, and 4,000 Hz. Listener sex was compared using a X^2^ test and all other group comparisons were made using an independent samples t-test*.

### Procedure

#### Audiometry and Hearing Aid Assessment

Hearing sensitivity was assessed for all children using age-appropriate behavioral audiometric assessment techniques. Children with normal hearing were screened via air conduction with headphones at 20 dB HL at 500, 1,000, 2,000, and 4,000 Hz. For children with hearing loss, air- and bone-conduction audiometric thresholds were measured at octave frequencies from 250 to 8,000 Hz using either Etymotic ER-3A insert or TDH-49 circumaural earphones. The thresholds at 500, 1,000, 2,000, and 4,000 Hz were averaged to calculate the pure-tone average (PTA) for each ear, and the PTA for the better-ear was used to represent degree of hearing loss in the statistical analyses.

For children with hearing loss, audibility for the long-term average speech spectrum for a 65 dB SPL input was measured at their daily use settings with the Audioscan Verifit probe microphone system (Dorchester, Ontario). The Verifit calculated the aided Speech Intelligibility Index (SII; ANSI S3.5-1997) for the 65 dB SPL speech signal for each ear as an estimate of speech audibility in quiet. The sensation level of the hearing aid output in 1/3 octave was measured and multiplied by an importance weight specified in the ANSI standard to represent the amount of speech information in each band. The weighted audibility across bands was added together to calculate the weighted proportion of speech that was audible through the child's hearing aids. The SII was expressed as a value between 0 and 1, where 0 indicates that none of the speech spectrum was audible, and 1 indicates that the entire speech spectrum was audible. Aided SII data for the children who wore hearing aids are shown in Table 1. The output of the hearing aid was measured in the child's ear canal whenever possible. If the child was uncooperative, the child's real-ear-to-coupler-difference (RECD) was measured for each ear. The RECD was then applied to measures of hearing aid output in the 2 cm^3^ coupler on the Verifit system to simulate the output of the hearing aid in the child's ear canal. The proximity of each child's fitting to Desired Sensation Level (DSL; Scollie et al., 2005) prescriptive targets at 500, 1,000, 2,000, and 4,000 Hz was measured and the geometric mean of the errors was taken to estimate the root-mean-square (RMS) error for each hearing aid. The average RMS error for each ear is reported in Table 1. Average hours of daily hearing aid use were assessed to describe the consistency of hearing aid use for children in the sample. Hearing aid use was estimated by either parent report or the automatic data logging system in the hearing aids. Because the hearing aids used by the children in the study were fitted by audiologists who were not associated with the study, information about specific signal processing features activated in the hearing aids from the fitting software were not available with one exception. Information about frequency lowering was collected. Frequency lowering (Phonak Sound Recover or Oticon Speech Rescue) was activated in 40% of the 1st grade fittings and 45% of the 3rd grade fittings.

#### Language, Working Memory, and Auditory Attention

Each child completed standardized measures of language, working memory, and executive function. Children with hearing aids used their hearing aids during these assessments. Receptive vocabulary skills were assessed using the Peabody Picture Vocabulary Test (PPVT-IV; Dunn and Dunn, 2007). Children were presented with target words and then pointed to a picture in a set of four possible pictures that best corresponds to the target word. Visuospatial working memory was assessed using the Odd-One-Out subtest of the Automated Working Memory Assessment (AWMA; Alloway et al., 2008). This visuospatial working memory task was selected to minimize the effects of differences in hearing and language abilities on working memory performance between children with normal hearing and children with hearing loss. For the Odd-One-Out, children are visually presented with sets of three complex shapes. One of the shapes is different than the other two shapes. The child points to the shape that does not match the other shapes. The child is then asked to remember the position of the different shape on a screen with three blank boxes. The number of sets of shapes increases throughout the task until the child misses a specific number of sets across consecutive blocks of trials. The PPVT and AWMA yielded raw scores and standard scores with a normative mean = 100 and a standard deviation = 15. Each child also completed the Auditory Attention subtest of the Developmental Neuropsychological Assessment (NEPSY-II; Brooks et al., 2009), which measured the ability to sustain auditory attention. During the Auditory Attention subtest of the NEPSY, children listen to a recorded series of words that are presented at a rate of one per second. The child must attend to the words and touch a red circle each time that they hear the word “red,” but not for other words. The total score is based on a combination of accuracy for “red” trials, where an incorrect response would be not touching the red circle when “red” was presented, and errors where the child touched the red button for any other word. The Auditory Attention subtest yields a scaled score with a normative mean = 10 and a standard deviation = 3.

#### Adaptive Speech Recognition Task

The stimuli for the speech recognition task were 250 low-predictability sentences described in a previous study (McCreery et al., 2017). Each sentence included four key words that were within the lexicon of 5-year-old children based on a child lexical database (Storkel and Hoover, 2010). The sentences were constructed with a simple, subject-verb-adjective-object syntactic structure. The sentences were recorded at 44,100 Hz sampling rate with 32-bit resolution as spoken by a female, native-English talker. An unmodulated speech-spectrum noise was created by taking the Fast Fourier Transform (FFT) of the concatenated set of sentences, randomizing the phase at each time point, and taking the inverse-FFT of the resulting signal to generate a noise that matched the long-term average spectrum of the talker. The noise had a cosine-squared 100 ms ramp-up before and ramp-down after each stimulus. For the simulated reverberation conditions, the target sentences and masker were convolved with the binaural room impulse response for a small classroom (20” × 20”) with a reverberation time of 600 ms (RT60), which was the modal reverberation time for a sample of classrooms in a study of classroom acoustics that included children in the age range of this experiment (Dockrell and Shield, 2006).

Children were seated in a sound-attenuating audiometric test room or mobile van with the examiner. The speech and noise were presented from two speakers co-located in the front of the child at a position where the speech was calibrated at 65 dB SPL using a 1,000 Hz calibration tone with the same RMS level as the speech stimuli. Presentation in sound field was used so children could listen through their hearing aids. Children with hearing loss listened to conditions at their normal hearing aid use settings. Sentences were chosen at random without replacement for each trial. The level of the masker was adapted using a one-down, one-up procedure (Levitt, 1971) with custom software to estimate the signal-to-noise ratio where each child got 50% of the sentences correct (SNR50). The starting SNR for each track was 20 dB, the initial step-size was 5 dB, and after two reversals, the step size decreased to 3 dB for the final 6 reversals. Because the stopping rule for the adaptive track was based on the number of reversals, the number of sentences presented for each track varied across children from 20 to 42 with an average of 25 sentences per condition. The examiner scored responses during the task. Noise and noise + reverberation conditions were completed by each child in random order.

#### Statistical Analyses

All statistical analyses and data visualization were completed using R Statistical Software (R Development Core Team, 2018). Data visualization was completed using the ggplot2 (Wickham and Chang, 2016) and sjPlot (Lüdecke and Schwemmer, 2018) packages for R. Descriptive statistics were generated for each predictor and outcome measure. For language and cognitive measures, standard scores were used to compare children in the experiment to the normative sample for each test, whereas raw scores were used to represent each construct in statistical analyses with age or grade as a covariate. Pearson correlations were calculated between predictors and outcomes to show the pattern of bivariate relationships between the predictors and outcomes for the study to support the inclusion of predictors in the multivariate models. For all the children, a linear mixed model was conducted to test the effects of linguistic and cognitive skills on speech recognition in noise and noise + reverberation using the lme4 package (Bates et al., 2015) for R. All possible interaction terms were assessed for each model, but only interactions that met the criterion for statistical significance (*p* < 0.05) are reported for simplicity with the exception of the subject type (NH vs. HL) interaction with reverberation, which was specifically hypothesized. The effects of aided audibility on language, working memory, and SNR50 in reverberation were also assessed for children with hearing loss with linear regression using a mediation analysis approach. The normality of each model's residuals was assessed to identify potential violations of statistical assumptions. To control for Type I error rate for statistical tests involving multiple comparisons, the *p*-values were adjusted using the False Discovery Rate procedure (Benjamini and Hochberg, 1995).

## Results

**Figure 1:**
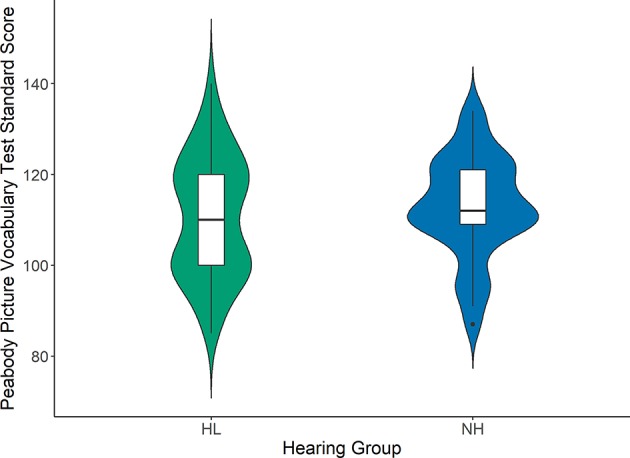
Peabody Picture Vocabulary standard scores for children with hearing loss (HL; green) and children with normal hearing (NH; blue). Box plots represent the median (middle line) and interquartile range of the data. The colored regions around each box blot are symmetrical representations of the distribution of data points in each condition.

**Figure 2:**
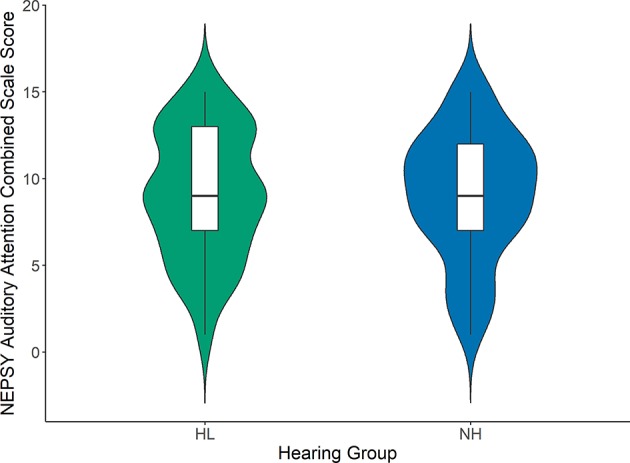
NEPSY II Auditory Attention combined scaled scores for children with hearing loss (HL; green) and children with normal hearing (NH; blue). Box plots represent the median (middle line) and interquartile range of the data. The colored regions around each box blot are symmetrical representations of the distribution of data points in each condition.

**Figure 3:**
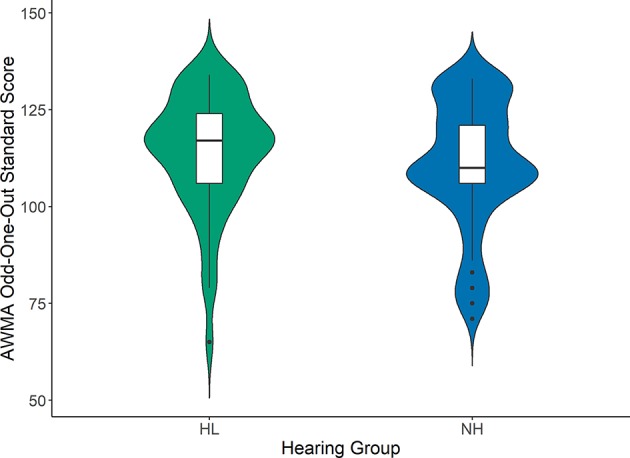
Automated Working Memory Assessment Odd-One-Out subtest standard scores for children with hearing loss (HL; green) and children with normal hearing (NH; blue). Box plots represent the median (middle line) and interquartile range of the data. The colored regions around each box blot are symmetrical representations of the distribution of data points in each condition.

Figures 1–3 compare the standard scores between children with hearing loss and children with normal hearing on the PPVT, NEPSY Auditory Attention, and AWMA Odd-One-Out tasks, respectively. There were no significant differences between children with hearing loss and children with normal hearing on these measures based on two-sample *t*-tests (see Table 1). Table 2 shows the Pearson correlations between the linguistic and cognitive standard scores and SNR50 for noise and the SNR50 for noise plus reverberation conditions for all participants. All of the predictor variables were significantly correlated with SNR50 for both speech recognition conditions. The strength of the significant correlations was medium (0.28) to large (0.80) for each bivariate relationship (Cohen, 1988).

**Table 2:** Pearson correlations between speech recognition, cognition, and linguistic factors for all children.

	**SNR50—N**	**SNR50—N+R**	**PPVT**	**NEPSY attention**	**AWMA OOO**
SNR50—N					
SNR50—N + R	0.801^*^				
PPVT	−0.460^*^	−0.452^*^			
NEPSY attention	−0.258^*^	−0.264^*^	0.243^*^		
AWMA	−0.324^*^	−0.309^*^	0.445^*^	0.332^*^	

SNR50, Signal-to-noise ratio for 50% correct; N, Noise; R, Reverberation; PPVT, Peabody Picture Vocabulary Test; NEPSY Attention, NEPSY Auditory Attention subtest; AWMA OOO, Automated Working Memory Assessment Odd-One-Out subtest

* *p < 0.05*.

**Figure 4:**
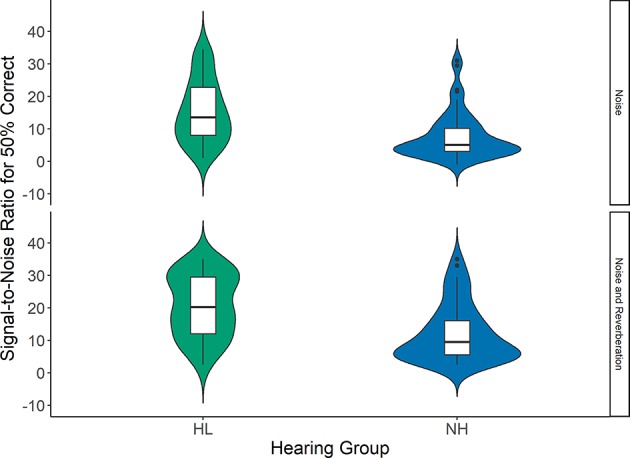
The signal-to-noise ratio (SNR) for 50% correct sentence recognition for children with hearing loss (HL; green) and children with normal hearing (NH; blue). The top panel shows data for noise, and the bottom panel shows data for noise + reverberation. Box plots represent the median (middle line) and interquartile range of the data. The colored regions around each box blot are symmetrical representations of the distribution of data points in each condition.

Figure 4 shows the SNR50 for both groups of children in noise and noise plus reverberation conditions. The effects of reverberation condition (noise and noise plus reverberation), grade (1st vs. 3rd), language (PPVT), auditory attention (NEPSY), visuospatial working memory (AWMA Odd-One-Out) on SNR50 for sentence recognition for children with normal hearing and children with hearing loss were examined using a linear mixed model. Table 3 shows the statistical results of that model. Children with normal hearing had an SNR50 (Mean = 7.7 dB) that was significantly lower, by 8.1 dB, than children with hearing loss (Mean = 15.8 dB). Reverberation significantly increased the SNR50 by 5.5 dB. The lack of a significant group by reverberation condition interaction indicates that the magnitude of the group differences did not vary significantly between noise and noise + reverberation conditions. Children in 1st grade had higher (3.1 dB) SNR50 than children in 3rd grade, but this difference was not significant after controlling for other factors. Children with better visuospatial working memory and receptive vocabulary had significantly lower SNR50 than children with lower scores in these domains. There was no statistically significant difference in SNR50 based on individual differences in auditory attention after controlling for other factors.

**Table 3:** Linear mixed model for predictors of SNR50 for all children.

**Predictors**	**Estimates**	**Confidence interval**	***p***
Subject type (NH vs. HL)	−8.1	−10.5 to −6.34	**<0.001**
Grade (1st vs. 3rd)	3.11	0.25 to 6.4	0.064
Reverberation (None vs. RT60 = 600 ms)	5.5	1.25 to 6.5	**<0.001**
NEPSY attention	−0.24	−0.65 to 0.30	0.226
AWMA OOO	−0.33	−0.59 to −0.07	**0.032**
PPVT	−0.25	−0.45 to −0.05	**<0.001**
Subject type x reverberation	−1.19	−1.98 to −0.24	0.41
**Random effects**			
Residual variance (σ^2^)	25.17		
Subject variance	31.24		

*PPVT, Peabody Picture Vocabulary Test; NEPSY Attention, NEPSY Auditory Attention subtest; AWMA OOO, Automated Working Memory Assessment Odd-One-Out subtest; Estimates represent the coefficients for each variable in the model. For categorical predictors, the estimate represents the mean difference. For continuous predictors, the estimate represents the change in SNR50 for a one unit change in the predictor. All p-values for significant effects are bolded*.

Table 4 includes the Pearson correlations between speech recognition, auditory variables (better-ear aided SII and better-ear pure tone average), and cognitive and linguistic standard scores for the children with hearing loss. Similar to the combined correlations for children with normal hearing and children with hearing loss (Table 2), the SNR50 for each condition was correlated with receptive vocabulary and working memory. Additionally, the NEPSY Auditory Attention score was correlated with the noise plus reverberation condition, but not the noise condition. Better-ear pure-tone average was not significantly associated with any predictor or outcome. The better-ear aided SII was correlated with receptive vocabulary, but not other predictors. The strength of the significant correlations was medium (0.32) to large (0.82) for each bivariate relationship (Cohen, 1988).

**Table 4:** Pearson correlation between speech recognition and auditory variables, cognition, and linguistic factors for children with hearing loss.

	**SNR50-Noise**	**SNR50–N+R**	**PPVT**	**NEPSY attention**	**AWMA OOO**	**Better-ear SII**	**Better-ear PTA**
SNR50 – Noise							
SNR50 – N + R	0.737^*^						
PPVT	−0.457^*^	−0.351^*^					
NEPSY attention	−0.222^*^	−0.245^*^	0.121				
AWMA OOO	−0.420^*^	−0.321^*^	0.323^*^	0.245^*^			
Better-ear aided SII	−0.210	−0.305^*^	0.325^*^	0.111	0.032		
Better-ear PTA	0.073	0.032	−0.206	−0.162	−0.092	−0.846^*^	

SNR50, Signal-to-noise ratio for 50% correct; N+R, Noise plus reverberation; PPVT, Peabody Picture Vocabulary Test; NEPSY Attention, NEPSY Auditory Attention subtest; AWMA OOO, Automated Working Memory Assessment Odd-One-Out subtest; Better-ear SII, Aided SII for 60 or 65 dB SPL speech signal; Better-ear PTA, Audiometric pure-tone average of thresholds at 500–4,000 Hz in the better ear

* *p < 0.05 (after adjustment for False Discovery Rate)*.

To examine the relationship between aided audibility, language, and speech recognition for children with hearing loss, a series of linear regression models were conducted with the children with mild-to-severe hearing loss to test whether the relationship between aided speech audibility and speech recognition in noise and reverberation was mediated by language skills. Table 5 includes the results from the models. Individual regression models with better-ear aided SII and PPVT as predictors of aided SNR50 in noise and reverberation were completed. Each model indicated that audibility and language were significant individual predictors of SNR50 for children with hearing loss. A combined model that included both language and audibility was conducted and yielded the same pattern of results as the individual models. This pattern of results suggests that aided audibility has a direct positive effect on the SNR50 for noise and reverberation and an indirect positive effect on SNR50 through language ability based on the linear regression model between better-ear aided SII and language for children with hearing loss who wear hearing aids.

**Table 5:** Linear regression models for the mediation effects of language and audibility on SNR50 in reverberation for children with hearing loss.

**Predictors**	**Estimates**	**Confidence interval**	***p***
**FULL MODEL**			
PPVT	−0.32	−0.15 to −0.55	**<0.001**
Better-Ear aided SII	−4.85	−3.21 to −5.34	**0.04**
**PATH MODELS**			
**Language predicting SNR 50-R**			
PPVT	−0.28	−0.10 to 0.42	**<0.01**
**Better-Ear aided SII predicting SNR 50-R**			
Better-Ear aided SII	−5.43	−3.4 to −7.1	**0.01**

*SNR50-R, SNR-50 for the reverberation condition; Better-Ear SII; PPVT, Peabody Picture Vocabulary Test; Estimates represent the coefficients for each variable in the model. For continuous predictors, the estimate represents the change in SNR50 for a one unit change in the predictor. All p-values for significant effects are bolded*.

## Discussion

The goal of this study was to measure aided speech recognition in noise and reverberation for children with hearing loss and a group of children with typical hearing matched for age and socioeconomic status. Children with hearing loss completed speech recognition in noise and noise + reverberation with their hearing aids. Auditory, cognitive, and linguistic factors were analyzed to determine if they predicted individual differences in speech recognition in noise and reverberation. For children with hearing loss, the inter-relationships between speech audibility, language, and speech recognition in noise and in noise + reverberation were also examined separately. As expected, children with hearing loss performed more poorly than children with normal hearing in noise and in noise plus reverberation conditions. Individual differences in speech recognition for children with normal hearing and children with hearing loss in all adverse conditions were partially predicted by language, working memory, and auditory attention. For children with hearing loss, the better-ear aided audibility for speech was a positive predictor of language and the aided SNR50 for noise + reverberation. Language also significantly predicted the aided SNR50 even after controlling for audibility.

Children with hearing loss were at a significant disadvantage when listening in adverse conditions compared to peers with typical hearing, even with amplification. Overall, children with normal hearing had an SNR50 that was more than 8 dB better than children with hearing loss. Reverberation (RT60) of 600 ms reduced SNR50 for both groups by an additional 5 dB. Although children with hearing loss performed more poorly than peers with normal hearing, the performance difference between groups was similar for both noise and noise plus reverberation conditions. The finding of poorer performance for children with hearing loss is consistent with the previous literature (Finitzo-Hieber and Tillman, 1978), but the previously observed pattern where reverberation disproportionately affected children with hearing loss was not replicated. We speculate that the interaction between hearing status and reverberation condition in the Finitzo-Hieber and Tillman study may have been driven by the fact that children with hearing loss in that study only listened monaurally. However, there are numerous other differences between the participants, amplification conditions, and experimental design of the studies that make it difficult to pinpoint why the interaction between hearing status and reverberation for speech recognition was not observed in this study. Future research could focus on further elucidating these factors.

As predicted by the ELU model (Rönnberg et al., 2008), individual cognitive and linguistic abilities were associated with speech recognition in noise and noise plus reverberation for children with hearing loss and children with normal hearing. Children with better vocabulary and working memory have better speech recognition in noise and noise plus reverberation conditions than peers with poorer skills in these areas. There were no interactions between these effects and condition, suggesting that the relationship between cognitive and linguistic abilities was similar for noise and noise plus reverberation conditions. These results extend previous research based on the ELU model to include children with hearing loss in conditions of noise and reverberation, which had not been examined previously. Further, the inclusion of auditory attention is consistent with predictions that listening in adverse conditions may be related to susceptibility to informational masking that can occur with reverberation (Durlach et al., 2003) and the disruption of temporal cues in the speech signal by noise and reverberation, as age-related changes in the ability to use temporal cues have been posited to be associated with attention (Hall et al., 2012).

Our previous research has also demonstrated that children with better aided speech audibility have better speech recognition under degraded conditions because of the direct effects of audibility on speech recognition (McCreery et al., 2015, 2017), as well as indirect effects due to the cumulative influence of audibility on language development (Tomblin et al., 2015). Separate linear regression analyses of children with hearing loss in the current study indicated that audibility was positively associated with language and speech recognition in noise and reverberation, but that language also had a unique contribution to speech recognition in degraded conditions. This pattern confirms the pattern from previous research for both direct and indirect associations between audibility and speech recognition in noise for children with hearing loss. Audibility not only benefits the child through signal audibility, but also through an accumulation of auditory experience over time that fuels the language skills needed to understand speech in adverse conditions. This finding highlights the importance of consistent hearing aid use for children with hearing loss to promote access to sound for speech recognition and for long-term development of the linguistic skills that support degraded speech recognition (Tomblin et al., 2015).

Despite the fact that this study was one of the first to examine the auditory, cognitive, and linguistic factors that predict speech recognition in noise and reverberation for children who wear hearing aids, there are several limitations that could be addressed in future research on this topic. The reverberation simulation used in this study was implemented to be completed with minimal equipment requirements so that children could be tested at multiple sites. More sophisticated methods of reverberation simulation have been developed and used in recent studies with adults with hearing loss (Zahorik, 2009; Reinhart and Souza, 2016) and children with normal hearing (Wróblewski et al., 2012) or cochlear implants (Neuman et al., 2012). Future studies should take advantage of these methods to complete a more realistic assessment of speech recognition in noise and reverberation than was possible in the current study. Measures of working memory and auditory attention were chosen that would be appropriate for children with hearing loss and minimize potential confounds related to differences in audibility and language skills across subjects. A visuospatial working memory task was used, but the auditory presentation of the attention task may have been affected by auditory or linguistic abilities. The measure of auditory attention showed weak relationships with language and audibility, but future research could include visuospatial attention tasks to further minimize potential confounds. The study design also did not include realistic masking or spatial conditions that children might encounter in their everyday listening environments, which have been examined in other studies of children with normal hearing (MacCutcheon et al., 2019) and children with hearing aids (Ching et al., 2011) or cochlear implants (Misurelli and Litovsky, 2012). Thus, we expect that children with hearing loss may have performed more favorably if the target and masker were spatially separated than in the current study where target and masker were co-located. However, previous research with children with hearing loss demonstrates large individual differences in spatial release from masking with hearing aids (Ching et al., 2011; Browning et al., 2019). Thus, the effects of noise and reverberation on aided spatial release from masking in children with hearing loss would need to be directly examined in future research.

The current study was conducted as part of a longitudinal study of children with mild to severe hearing loss, and therefore, did not include children with cochlear implants. Children with cochlear implants are likely to have significant challenges in noise and reverberation (Neuman et al., 2012). The factors that predict individual differences in speech recognition in adverse conditions in that population should be examined in future studies. Previous studies with adults with hearing loss suggest that amplification parameters may influence speech recognition in noise and reverberation (Shi and Doherty, 2008; Reinhart and Souza, 2016); however, this study was conducted with children using their hearing aids at their personal use settings, and so amplification parameters were not manipulated. Individual differences in amplitude compression settings or other hearing aid signal processing features among children in the study may have contributed to individual variability in speech recognition scores. However, this study was not designed to assess the influence of amplification parameters or hearing aid signal processing features other than audibility on speech recognition in degraded conditions. Future studies could include manipulation of children's amplification parameters to address this question.

## Data Availability Statement

The datasets generated for this study are available on request to the corresponding author.

## Ethics Statement

The studies involving human participants were reviewed and approved by Boys Town National Research Hospital Institutional Review Board. Written informed consent to participate in this study was provided by the participants' legal guardian/next of kin. All children completed written assent or consent to participate in the study.

## Author Contributions

RM, EW, MS, MB, and DL planned the proposed experiment. EW, MS, and MB were involved in data collection. RM wrote the first draft of the article. RM, EW, MS, MB, and DL edited and approved the final version of the article.

### Conflict of Interest

DL is a member of the Phonak Pediatric Advisory Board, but this work was not supported or affected by her involvement. The remaining authors declare that the research was conducted in the absence of any commercial or financial relationships that could be construed as a potential conflict of interest.
